# Supplementary material for: Role and mechanism of NCAPD3 in promoting malignant behaviors in gastric cancer
Source: Front Pharmacol. 2024 Apr 22;15:1341039. doi: 10.3389/fphar.2024.1341039 (PMC11070777; doi:10.3389/fphar.2024.1341039)
Supplement: Supplementary file 11 [file DataSheet2.ZIP › GSEA/Canonical pathways/my_analysis.Gsea.1599462267220/REACTOME_TRANSLATION.html]

Details for gene set REACTOME\_TRANSLATION[GSEA]

|  || Dataset | filtered\_dataset.sample\_info.cls#WT\_versus\_NCAPD3\_MUT |
| Phenotype | sample\_info.cls#WT\_versus\_NCAPD3\_MUT |
| Upregulated in class | WT |
| GeneSet | REACTOME\_TRANSLATION |
| Enrichment Score (ES) | 0.23216762 |
| Normalized Enrichment Score (NES) | 0.9691875 |
| Nominal p-value | 0.49137932 |
| FDR q-value | 0.9184563 |
| FWER p-Value | 1.0 |
Table: GSEA Results Summary

  

Fig 1: Enrichment plot: REACTOME\_TRANSLATION      
 Profile of the Running ES Score & Positions of GeneSet Members on the Rank Ordered List

  

| SYMBOL | TITLE | RANK IN GENE LIST | RANK METRIC SCORE | RUNNING ES | CORE ENRICHMENT || 1 | 2935 | GSPT1 | 5 | 1.156 | 0.1169 | Yes |
| 2 | 92935 | MARS2 | 143 | 0.741 | 0.0956 | Yes |
| 3 | 54516 | MTRF1L | 187 | 0.687 | 0.1363 | Yes |
| 4 | 6191 | RPS4X | 196 | 0.682 | 0.2016 | Yes |
| 5 | 25973 | PARS2 | 266 | 0.620 | 0.2167 | Yes |
| 6 | 92399 | MRRF | 347 | 0.569 | 0.2184 | Yes |
| 7 | 78988 | MRPL57 | 557 | 0.441 | 0.1142 | Yes |
| 8 | 6208 | RPS14 | 604 | 0.421 | 0.1249 | Yes |
| 9 | 55157 | DARS2 | 686 | 0.385 | 0.1069 | Yes |
| 10 | 6182 | MRPL12 | 729 | 0.359 | 0.1141 | Yes |
| 11 | 79590 | MRPL24 | 800 | 0.320 | 0.0972 | Yes |
| 12 | 1973 | EIF4A1 | 804 | 0.319 | 0.1282 | Yes |
| 13 | 51373 | MRPS17 | 817 | 0.306 | 0.1515 | Yes |
| 14 | 6183 | MRPS12 | 838 | 0.280 | 0.1663 | Yes |
| 15 | 6301 | SARS | 856 | -0.259 | 0.1810 | Yes |
| 16 | 7453 | WARS | 900 | -0.325 | 0.1840 | Yes |
| 17 | 16 | AARS | 921 | -0.349 | 0.2060 | Yes |
| 18 | 1978 | EIF4EBP1 | 938 | -0.362 | 0.2322 | Yes |
| 19 | 833 | CARS | 1022 | -0.420 | 0.2162 | No |
| 20 | 1983 | EIF5 | 1219 | -0.595 | 0.1373 | No |
Table: GSEA details [plain text format]

  

Fig 2: REACTOME\_TRANSLATION      
 Blue-Pink O' Gram in the Space of the Analyzed GeneSet

  

Fig 3: REACTOME\_TRANSLATION: Random ES distribution      
 Gene set null distribution of ES for **REACTOME\_TRANSLATION**
